# NhaD type sodium/proton-antiporter of *Halomonas elongata*: a salt stress response mechanism in marine habitats?

**DOI:** 10.1186/1746-1448-2-10

**Published:** 2006-07-27

**Authors:** Matthias Kurz, Anika NS Brünig, Erwin A Galinski

**Affiliations:** 1 Institut für Mikrobiologie und Biotechnologie, Rheinische Friedrich Wilhelms-Universität Bonn, Meckenheimer Allee, Bonn, Germany

## Abstract

**Background:**

Sodium/proton-antiporters (Nha) are known to play an important role in pH- and Na^+^-homeostasis. In microorganisms several types with different capacity, affinity and selectivity for Na^+ ^and Li^+ ^exist. The homeostasis system of *E. coli*, NhaA and NhaB, is well researched, but the function of other types of Na^+^/H^+^-antiporters like NhaD is yet to be fully understood. Since several antiporters play an important role at various points in the physiology of higher organisms, one can speculate that the main functions of some of those procaryotic antiporters differ from pH- and Na^+^-homeostasis.

**Results:**

This study investigates the function and regulation of a gene encoding for a NhaD type antiporter which was discovered in the halophilic eubacterium *Halomonas elongata*.

The deduced primary amino acid sequence of the abovementioned gene showed more than 60% identity to known antiporters of the NhaD type from *Alkalimonas amylolytica*, *Shewanella oneidensis *and several other marine organisms of the γ-*Proteobacteria*. Evidence was found for a dual regulation of *H. elongata *NhaD expression. The gene was cloned and expressed in *E. coli*. Antiporter deficient NaCl and LiCl sensitive *E. coli *mutants EP432 and KNabc were partially complemented by a plasmid carrying the *H. elongata nhaD *gene. Surprisingly the LiCl sensitivity of *E. coli *strain DH5α having a complete homeostasis system was *increased *when NhaD was co-expressed.

**Conclusion:**

Since NhaD is an antiporter known so far only from halophilic or haloalcaliphilic *Proteobacteria *one can speculate that this type of antiporter provides a special mechanism for adaptation to marine habitats. As was already speculated – though without supporting data – and substantiated in this study this might be active Na^+^-import for osmoregulatory purposes.

## Background

*Halomonas elongata *is a moderately halophilic eubacterium which thrives under a broad range of salinities [1] and has its growth optimum at 3% NaCl (w/v) (500 mM) [2]. The organism is known for the production of the compatible solutes ectoine and hydroxyectoine, which are accumulated for adaptation to high environmental salinity and correspondingly high osmotic activity [3,4].

In *Bacteria *adaptation of intracellular to extracellular osmotic activity is also coupled to a range of physiological properties like internal pH [5] and ionic strength [6], but also heat- and cold-tolerance. Especially Na^+^- and pH-homeostasis play an important role in physiology and are closely linked. Since sodium/proton-antiporters have a central function in both processes they are found in the cytoplasmic membranes of almost all cells [7,8] with *Clostridium fervidum *being the only known exception so far [9].

According to present knowledge, in *Bacteria *Na^+^/H^+^-antiporters serve three main functions:

1) Conversion of a proton gradient into a sodium gradient. This is necessary for the import of Na^+^-co-transported substrates and function of some flagellar motors.

2) Discharging of Na^+ ^and Li^+ ^from the cytoplasm.

3) Regulation of internal pH in alkaline environments.

As reviewed [10] a system consisting of a high capacity, low affinity type Na^+^/H^+^-antiporter NhaA in concert with a low capacity, high affinity antiporter, NhaB [11], is sufficient for bacteria from a diversity of environments to adapt to a broad spectrum of moderate salinities. Even then NhaA is the key system, NhaB being only essential when NhaA is absent. *E. coli *Na^+^/H^+^-antiporters NhaA and NhaB are well researched model systems [7] and several antiporter deletion mutants are at hand [12,11,13].

The exact role of Na^+^/H^+^-antiporters of other types is not completely understood so far. For example in the marine human pathogen *Vibrio cholerae *wildtype Na^+^- and Li^+^-tolerance is only displayed when all four different antiporters (NhaA, NhaB, NhaC and NhaD) and a NADH-quinon-oxidoreductase are present [14]. Conserved polar residues of *V. cholerae *NhaD antiporters have been identified and their function analyzed [15]. NhaD is also found in other pathogenic species but not in non-pathogens from the same phyla [16] which might rise speculations of NhaD being a pathogenicity marker. Recent research into NhaD type antiporter from *Alkalimonas amylolytica *revealed that this antiporter helps in adaptation to alkaline environments [17], thus contradicting the idea that NhaD might be a marker for pathogens. A paradoxical mode of NhaD operation suggests that NhaD type antiporters might be part of regulation in adaptation to saline environments.

For *Halomonadaceae *no Na^+^/H^+^-antiporters were known so far. Finding an NhaD type antiporter in the moderately halophilic non-pathogenic *H. elongata *adds further proof to the idea, that this type is rather involved in (marine) osmoregulation.

## Results

### NhaD sequence

**Figure 1:**
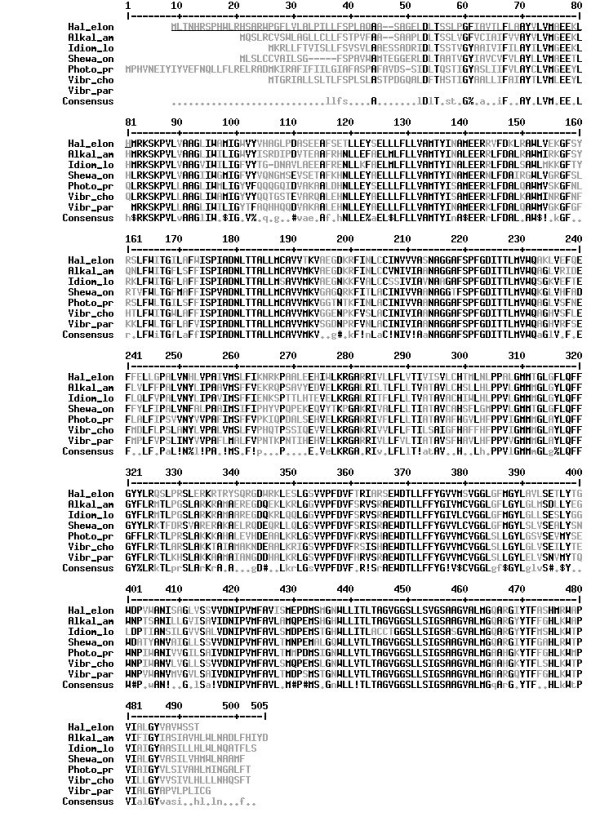
**sequence Alignment**. Multi sequence alignment of *H. elongata *NhaD and NhaD type antiporters of other γ-proteo bacteria. Hal_elon *Halomonas elongata*, Alkal_am *Alkalimonas amylolytica*, Idiom_lo *Idiomarina loihiensis*, Shewa_on *Shewanella oneidensis*, Photo_pr *Photobacterium profundum*, Vibr_ch *Vibrio cholerae*, Vibr_par *Vibrio parahaemolyticus*. Alignment was done with the MULTALIN [32] sequence tool. Spans with high homology (at least six of the seven organisms show the same residue) are given in black, spans with low or no homology in gray. Similar residues are noted in the consensus sequence as follows: ! is I, V; $ is L, M; % is F, Y; # any one of N, D, Q, E, B, Z. Underlined residues in the *H. elongata *sequence are probably not expressed *in vivo *and are not expressed in the reconstitution experiments.

**Figure 2:**
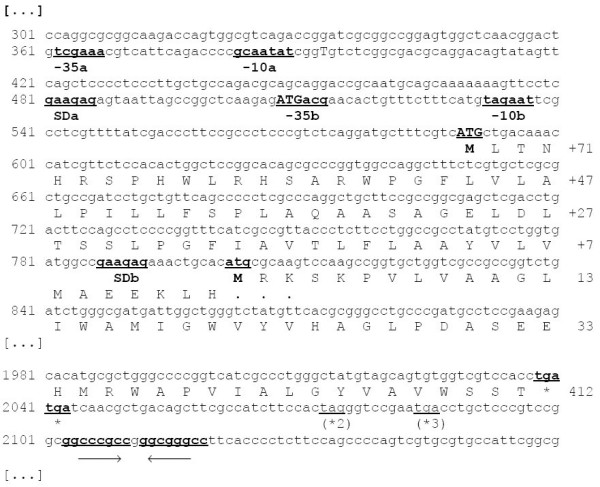
**H. elongata nhaD sequence**. Part of the DNA sequence of *H. elongata nhaD *and regions *upstream *and *downstream *of the coding region: Bases are numbered on the left (numbering according to full sequence as found in EMBL nucleotide sequence database: AM167899), the deduced AA sequence is below the codons and numbered on the right. **-35a**, **-10a **putative *rpo*H dependent promoter, **SDa **putative Shine-Dalgarno sequence after *rpo*H and two putative start codons **ATG**. **-35b **and **-10b **putative σ70 housekeeping promoter, **SDb **alternative Shine-Dalgarno sequence and **atg**possible start codon. Note that the last start codon is the only one usable for expression under control of both putative promoters. **tga**stopp codons in the first *, second *2 and third *3 reading frame. →← *inverted repeat*, the downstream sequence lacks an AT-rich motive, therefore this is probably not significant for transcription termination. Note also that there are two possible starting points for transcription and translation, yielding proteines of 483 or 412 residues. Translation starting after SDb would yield only the 412 AA protein. On the other hand SDa would only be functional only for the *rpo*H dependent promoter.

Using wobble primers in a touchdown PCR on *H. elongata *genomic DNA we discovered a DNA sequence with a high similarity to the translation product of *Vibrio parahaemolyticus nhaD*, a novel type sodium/proton antiporter [12]. Using standard chromosome walking techniques and inverted PCR we acquired a sequence approximately 2800 base pairs (bp) long (full sequence available at the EMBL nucleotide sequence database, AM167899). The sequence contains at least one open reading frame (ORF) preceeded by a Shine-Dalgarno sequence (SD) [18]. The ORF was designated *nhaD *due to the high similarity of the 412 amino acid (AA) transcription product with NhaD type antiporters of several marine γ-*Proteobacteria *(Fig. 1). Two putative promoter regions were found upstream of the ORF (Fig. 2). One of them is similar to *E. coli *σ *70 *type *housekeeping *promoters and the other one resembles a *rpoH *dependent *heat shock *promoter (Tab. 1).

**Table 1:** Promoter comparison

promoter	Organism	-35 region	Spacer	-10 region
σ70 *nhaD *putative	*H. elongata*	aTGACg	18	TAgAAT
σ70 *ectA *putative	*H. elongata*	TTGAaA	18	TATgAT
σ70 consensus	*E. coli*	TTGACA	16–18	TATAAT
*rpo*H *nhaD *putative	*H. elongata*	tcGAAA	15	gCaATaT
*rpo*H consensus	*E. coli*	CTGAAA	11–16	CCCATnT

Comparison of putative *H. elongata *promotor elements and *E. coli *consensus sequences for σ70 and *rpoH *dependent promoters: The putative σ70 *ectA *promoter was found by Göller [24], and recent umpublished Data from our group). Consensus sequences are according to the review of Wösten [23]. Bases identical to the consensus sequence are given in upper case, differing bases in lower case. Note that the undefined nucleotide (n) in the *rpoH *consensus and its equivalent are also given in lower case.

Between the putative *rpo*H and σ*70 *dependent promoters a second SD was detected. An ORF using the same reading frame would yield a putative alternate NhaD product which has an N-terminus of additional 71 AAs and would yield a total of 483 AAs.

The putative 412 AA protein has a calculated Mr of 45.7 kDa and a pI of 8.52. In the sequence 48 residues are basic, 22 are acidic and another 42 are uncharged polar, yielding a total of 112 (27%) hydrophilic and 300 (73%) hydrophobic residues. This complies with the overall composition of integral membrane proteins. The most abundant residues are 44 Leu (13%), 41 Val (10%) and 38 Ala (9.2%). Least abundant are Cys (4/1%), His (5/1.2%) and Gln (6/1.5%). Hydropathy plots show 10 to 12 possible transmembrane regions (data not shown). The data are very similar to those from *V. parahaemolyticus *NhaD [12] and support the concept that our protein is of the NhaD type.

### NhaD homologues

A BLAST search yielded seven bacterial sodium/proton antiporters of the NhaD type from Proteobacteria: *Alkalimonas amylolytica *(AY962404), *Shewanella oneidensis *(AE015538, putative), *Idiomarina loihiensis *(AE017340, putative), *Photobacterium profundum *(CR378677, putative), *Vibrio parahaemolyticus *(AB006008), *Vibrio cholerae *(AF331042) and *Vibrio vulnificus *(AE016812 – all accession numbers taken from NCBI database). Several additional Na^+^/H^+^-antiporters and transport proteins for other metals show significantly lower similarities.

Multiple sequence alignment with NhaD type antiporters revealed 297 identical residues (70% based on the 412 residues of the *H. elongata *protein) and 311 highly conserved residues (76%) (Fig. 2). All of those organisms are γ-proteobacteria, six of marine origin and one soda lake isolate, *A. amylolytica*.

We also gained part of a DNA sequence of haloalkaliphilic Lake Bogoria Isolate 25B1 [19], supposedly a member of the Family *Halomonadaceae*, which shows 95% identity to *H. elongata nhaD *on DNA level and therefore has equally high homology in the putative AA sequence (data not shown).

### Complementation of *E. coli *EP432 and Knabc

**Figure 3:**
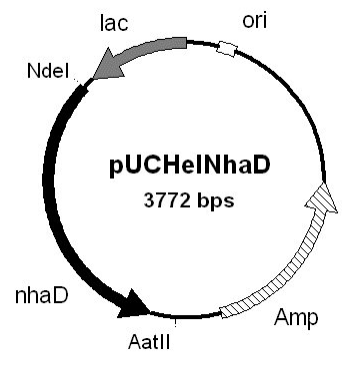
**Expression vector**. Expression vector pUCHelNhaD. *H. elongata nhaD *(nhaD) was amplified by PCR using mutated primers that integrate the *nhaD *start codon in a constructed Nde I restriction site. The gene was subsequently cloned between the Nde I and Aat II enzyme sites of pUC18. The pUC18 β-lactamase gene is destroyed by this insertion, and in frame replaced by *nhaD*. Expression of NhaD is controlled by the lac operator (lac). Amp depicts the gene encoding for ampiciline resistance, ori marks the *E. coli *replication origin.

We constructed an expression vector from pUC18 where the β-lactamase is replaced *in-frame *by *nhaD *and named it pUCHelNhaD (Fig. 3). We decided to use the ORF yielding a 412AA product for two reasons: First dual control (see below) indicates that two promoters might be involved in *nhaD *expression, one being a stress promoter. The putative σ*70 housekeeping *promoter (Fig. 2) would yield this (shorter) protein. Secondly, our product is analogous to the functional *V. parahaemolyticus *NhaD described by Nozaki and coworkers [12]. This is especially true for the ATG start codon.

*E. coli *mutants EP432 (*nhaA *and *nhaB *deleted *in frame*) and KNabc (*nhaA*, *nhaB *and *chaA *destroyed by insertion of omega cassettes) were transformed with pUCHelNhaD (complemented strain) and pUC18 (control). NhaD expression was under control of the pUC18 *lac *promoter. We decided to use both strains to assess possible differences in complementation by NhaD due to method of deletion and influence of the sodium extrusion system ChaA.

**Figure 4:**
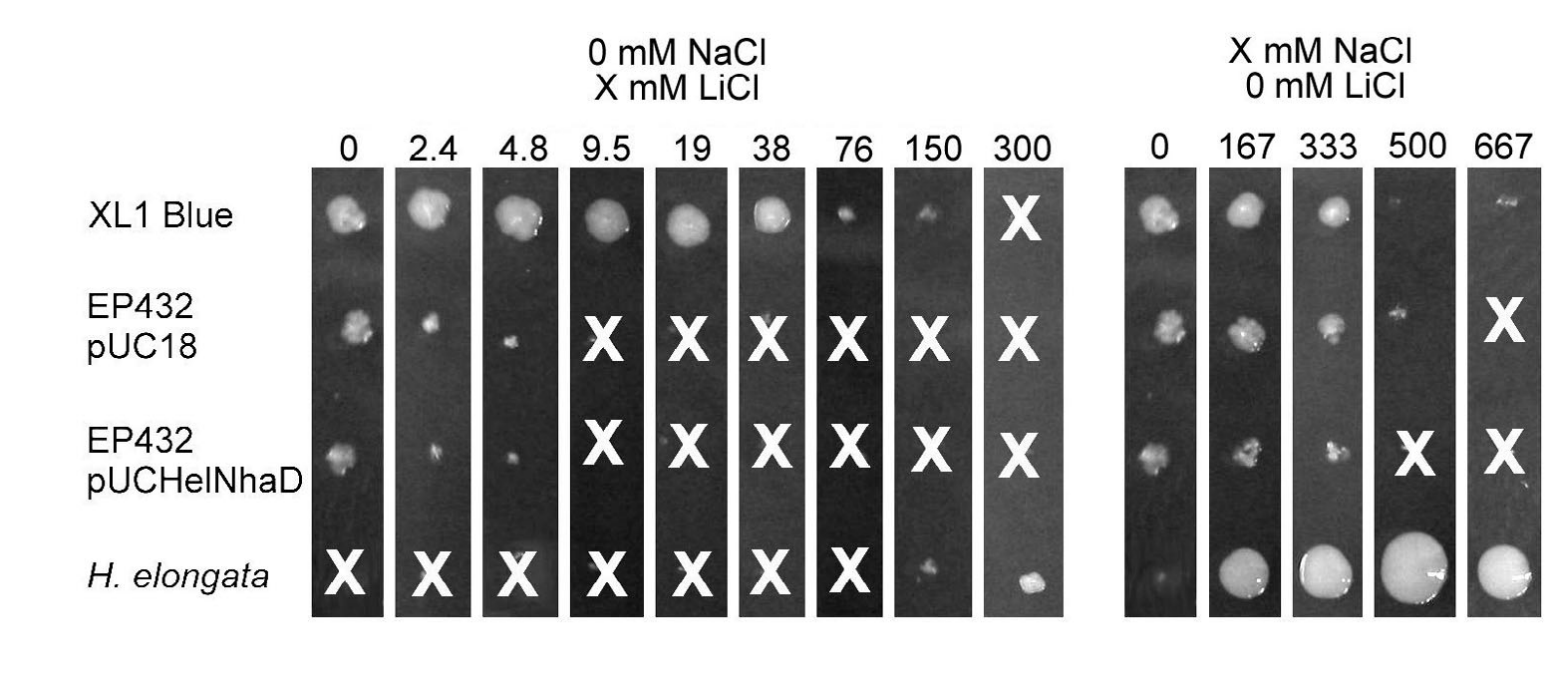
**Growth on solid minimal agar**. Colonies of *E. coli *strains XL1 Blue, EP432 pUC18 (control) and EP432 pUCHelNhaD and also of *H. elongata *were grown on MM63 minimal agar supplied with trace elements and vitamins for two days at 37°C. First set of pictures shows growth without NaCl (< 0.01% NaCl, 2 mM) due to media component impurities) under increasing LiCl concentrations, second set of pictures is growth under different salinities in the absence of LiCl. Concentrations of NaCl and LiCl are indicated above individual bars. No growth is marked with a white X (some artifacts due to toothpick transfer are visible). LiCl tolerance is drastically reduced in EP432 and this is not clearly counteracted by the presence of pUCHelNhaD although we observed slightly larger colonies. Salt tolerance is also reduced in this organism and only poorly compensated by expression of *H. elongata *NhaD. The halophilic *H. elongata *does not grow without salt and a corresponding osmotic activity in the growth medium. Apparently LiCl has no lethal effect on *H. elongata *at concentrations of 150 and 300 mM in solid medium and seems to partially replace the organism's sodium requirements.

First we tested growth on solid minimal medium and compared colony size of both mutants with *E. coli *XL1-Blue and *H. elongata *(Fig. 4). In the absence of NaCl (traces from media components available) XL1blue grew well with LiCl concentrations below 38 mM. Growth was also observed at up to 150 mM, but colonies are drastically smaller. Complemented EP432 and the control both grew well only without LiCl, and did not show growth at concentrations higher than 4.8 mM. In sharp contrast to this *H. elongata *did not grow at all with less than 150 mM LiCl and only traces of NaCl available.

*E. coli *wild type is known to grow on minimal medium containing up to to 3% NaCl (w/v) (500 mM) and, in the presence of solutes, to tolerate salinities up to 5% (w/v) (666 mM). On MM63 supplemented with 0.1% (w/v) yeast extract we observed growth for XL1-Blue but it was very poor at 3 and 4% NaCl (w/v) (500 and 666 mM). At 1% and 2% (w/v) (166 and 333 mM) the complemented EP432 strain and the control did grow, but colonies were smaller than those of XL1-Blue with the control colonies being even slightly smaller. At 3% (w/v) (500 mM) the complemented strain showed weak growth where the control did not.

**Figure 5:**
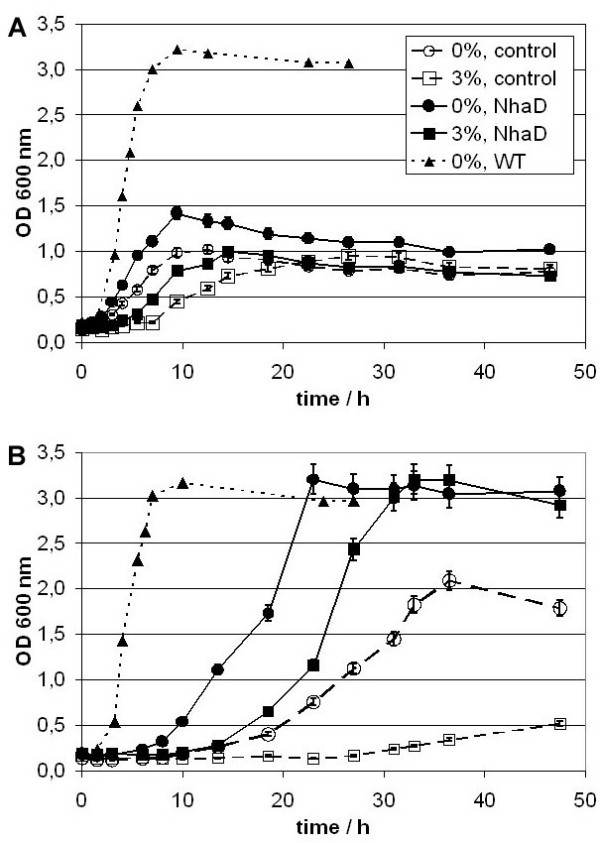
**Growth of *E. coli *EP432**. Growth in liquid Medium: *E. coli *strains EP432 pUC18 (open, control) and EP432 pUCHelNhaD (solid, NhaD) were grown at 0% ^1) ^(circles) and 3% (500 mM, squares) salinity (w/v) in A) LB medium and B) MM63 minimal medium. Additionally growth data for XL1 Blue (WT, triangles) are shown for 0%. ^1) ^Due to impurities of media components there are still traces of NaCl (<0.01%, 2 mM) in the medium.

**Figure 6:**
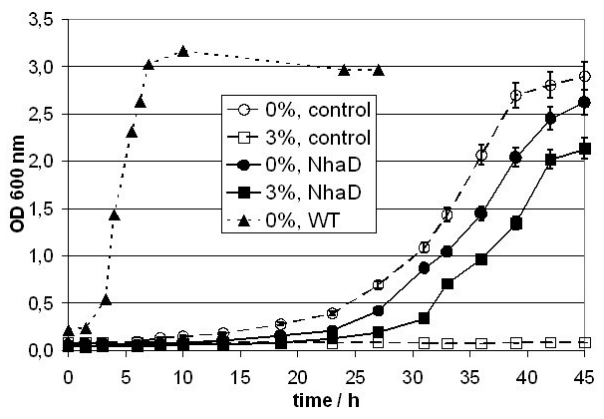
**Growth of *E. coli *Knabc**. Growth in liquid Medium: A) Growth curves of *E. coli *strains KNabc pUC18 (open Symbols, control) and KNabc pUCHelNhaD (black Symbols, NhaD) at 0% ^1) ^(circles) and 3% (500 mM, squares) salinity (w/v) in MM63 minimal medium. Additionally growth data for XL1 Blue (WT, triangles) are shown for 0%. ^1) ^Due to impurities of media components are still traces of NaCl (< 0.01%, 2 mM) in the medium.

When grown in liquid medium (Fig. 5 and 6, Tab. 2 and 3) enhanced growth was observed for complemented EP432 pUCHelNhaD and KNabc pUCHelNhaD compared to the controls carrying pUC18. We observed higher growth rates, shorter lag phases, higher OD maxima or a combination of those factors. Growth enhancement was more drastic in minimal medium (Fig. 5B). Interestingly, in complex medium (Fig. 5A) growth was comparably poor. We attribute this to the high content of Na^+^-co-transported substrates (see discussion). Even in the presence of only traces of NaCl, growth of the transport deficient EP432 was reduced compared to the complemented strain. The observed worse growth in KNabc compared to EP432 was to be expected since the latter strain still has the ChaA Na^+^-extrusion system.

**Table 2:** Growth of *E. coli *EP432

Culture	Strain	growth rate/h^-1^	rel. growth	lag phase/h	OD600 max
LB, 0% NaCl	NhaD	0.36 ± 0.05	1.4	2	0.85 ± 0.1
	Control	0.26 ± 0.05	1	2	0.65 ± 0.1
LB, 3% NaCl	NhaD	0.23 ± 0.05	1.5	4	0.65 ± 0.1
	Control	0.15 ± 0.05	1	6	0.65 ± 0.1
MM63, 0% NaCl	NhaD	0.22 ± 0.05	2.2	6	3.1 ± 0.2
	Control	0.10 ± 0.05	1	13	2.1 ± 0.2
MM63, 3% NaCl	NhaD	0.16 ± 0.05	2.2	10	3.2 ± 0.2
	Control	0.07 ± 0.05	1	31	2.0 ± 0.2

Growth in liquid Medium: *E. coli *strains EP432 pUC18 (control) and EP432 pUCHelNhaD (NhaD) were grown at 0% ^1) ^and 3% (500 mM) salinity (w/v) in LB medium and MM63 minimal medium. Growth rates, duration of lag phase and maximum OD(600 nm) depending on culture and strain are summarized. Relative growth rate is given for complemented strain compared to the control at same salinity.

^1) ^Due to impurities of media components there are still traces of NaCl (<0.01%, 2 mM) in the medium.

**Table 3:** Growth of *E. coli *Knabc

Culture	Strain	growth rate/h^-1^	rel. growth	lag phase/h	OD_600 _max
MM63, 0% NaCl	NhaD	0.14 ± 0.02	1.1	7	2.6 ± 0.2
	Control	0.13 ± 0.02	1	5	2.8 ± 0.2
MM63, 3% NaCl	NhaD	0.12 ± 0.02	n.a.	10	2.1 ± 0.2
	Control	n.a.	1	n.a.	n.a.

Growth in liquid Medium of *E. coli *strains KNabc pUC18 (control) and KNabc pUCHelNhaD (NhaD) at 0%^1) ^and 3% (500 mM) salinity (w/v) in MM63 minimal medium. Growth rates, duration of lag phase and maximum OD(600 nm) depending on culture and strain are summarized. Relative growth rate is given for complemented strain compared to the control at same salinity. It can not be calculated for 3% (500 mM).

^1) ^Due to impurities of media components are still traces of NaCl (< 0.01%, 2 mM) in the medium.

LiCl tolerance was also tested in shaking cultures. According to the data on solid medium no growth was observed at more than 4.8 mM LiCl. Below this concentration no significant differences in growth were observed between EP432 pUCHelNhaD and EP432 pUC18 or KNabc pUCHelNhaD and KNabc pUC18; this is true for pH values ranging from 7 to 9 (data not shown). We also tried to complement the NhaA deficient *E. coli *strain NM81 but results were poorly reproducible (not shown).

### Li^+^-sensitivity of *E. coli *DH5α

**Figure 7:**
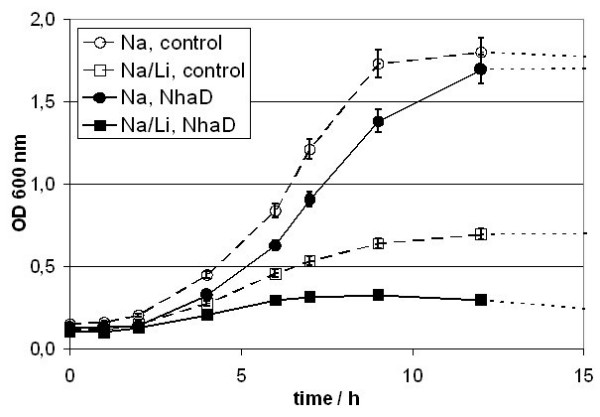
**Li^+^-sensitivity of E. coli DH5α**. A) Growth curves of E. coli strains DH5α pUC18 (open Symbols, control) and DH5α (black Symbols, NhaD) at 500 mM (3% (w/v)) NaCl (circles) and 300 mM NaCl/200 mM LiCl (squares) in MM63 minimal medium. Additionally growth data for XL1 Blue (WT, triangles) are shown for 0% salinity.

*H. elongata *is able to tolerate Li^+^-concentrations of up to 3 M depending on pH (Tab. 4). Therefore, *E. coli *DH5α where the full set of Na^+^-extrusion systems including NhaA, NhaB and ChaA is present was transformed with pUCHelNhaD and pUC18 (control). Both strains were grown at 500 mM (3% (w/v)) NaCl and at 300 mM NaCl/200 mM LiCl to test for enhanced Li^+ ^tolerance. As can be seen in Fig. 7 and Tab. 5, in the absence of LiCl there appeared to be only little difference in growth between the two strains and in the presence of LiCl growth of the control is inhibited. To our surprise expression of *H. elongata *NhaD did not increase LiCl tolerance in an *E. coli *having a functional set of Na^+^/H^+ ^antiporters. In contrast to our expectations next to no growth was observed in *E. coli *DH5α pUCHelNhaD, thus clearly indicating that NhaD is able to import Li^+^-ions into the cells. The same observations were made at a pH of 8.5 (in contrast to pH 7.3 of MM63) which is close to the average marine pH (data not shown). As can be seen in Tab. 6 lithium sensitivity is higher for the NhaD producing DH5*a *throughout the whole spectrum of pH and Li^+ ^concentrations used. Regrettably growth rates are not well reproducible since the cells tended to aggregate at low phosphate and higher Li^+ ^concentrations.

**Table 4:** Li^+^-tolerance of *H. elongata*

Mm Na^+^/mM Li^+^	pH 7	pH 7.5	pH 8	pH 8.5	pH 9
500/0	+++	+++	+++	+++	+++
400/100	+++	+++	+++	+++	+++
300/200	+++	+++	+++	+++	+++
200/300	+++	+++	+++	+++	+++
100/400	+++	+++	++	++	++
0/500	+++	+++	++	++	++
					
2000/0	+++	+++	+++	+++	+++
1500/500	+++	+++	++	++	++
1000/1000	++	++	+	+	+
500/1500	++	++	+	+	-
100/2000	+	+	+	-	-
100/2500	+	(+)	-	-	-
100/3000	(+)	(+)	-	-	-

Growth of *H. elongata *on solid and in liquid MM63 minimal medium, low-phosphate version supplemented with NaCl and LiCl as indicated. Growth quality judged from colony size and microscopic behaviour: +++ very good, ++ good, + poor, (+) survival, - no growth.

**Table 5:** Li^+^-sensitivity of *E. coli *DH5α

Culture	Strain	growth rate/h^-1^	rel. growth	lag phase/h	OD_600 _max
MM63, NaCl	NhaD	0.37 ± 0.03	1	2	2.8 ± 0.2
	Control	0.35 ± 0.03	1	2	3.0 ± 0.2
MM63, NaCl/LiCl	NhaD	0.20 ?	0.7	?	0.3 ?
	Control	0.28 ± 0.03	1	2	0.7 ± 0.1

Growth of *E. coli *strains DH5α pUC18 (control) and DH5α pUCHelNhaD (NhaD) at 500 mM (3% (w/v)) NaCl and 300 mM NaCl/200 mM LiCl in MM63 minimal medium. Growth rates, duration of lag phase and maximum OD(600 nm) depending on culture and strain are summarized. Relative growth rate is given for expression strain compared to the control at same salinity.

**Table 6:** pH dependend Li^+^-sensitivity of *E. coli *DH5α

MM Na^+^/mM Li^+^	pH 7	pH 7.5	pH 8	pH 8.5	pH 9
pUC18 control					
500/0	+++	+++	++	+	-
400/100	++	++	+	+	-
300/200	+	+	+	(+)	-
200/300	-	-	-	-	-
pUCHelNhaD					
500/0	+++	+++	++	+	-
400/100	++	++	+	(+)	-
300/200	+	+	(+)	-	-
200/300	-	-	-	-	-

Growth of *E. coli *strains DH5α pUC18 and DH5α pUCHelNhaD on solid and in liquid MM63 minimal medium, low-phosphate version supplemented with NaCl and LiCl as indicated. Growth quality judged from colony size and microscopic behaviour: +++ very good, ++ good, + poor, (+) mere survival of cells, - no growth.

### Expression analysis

**Figure 8:**
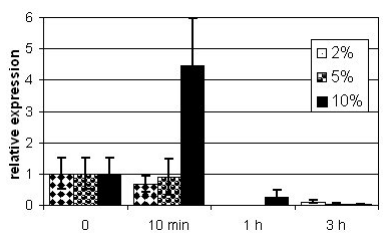
**NhaD expression in *H. elongata***. *nhaD *expression in *H. elongata *in complex Medium at different salinities: An overnight culture of *H. elongata *in K complex medium (5% salinity (w/v) (500 mM)) was transferred to the same medium at 2% (diamonds, 333 mM), 5% (circles, 833 mM) and 10% (1666 mM) salinity (w/v) (triangles/solid column) giving hypoosmotic, equiosmotic and hyperosmotic conditions respectively. Errors for mean of triplicate measurements is given. While growth curves and growth rates are similar, *nhaD *expression differs drastically for different media. After 10 minutes (lag-phase) expression levels are the same under hypo- and equiosmotic conditions. It increases by a factor of four under hyperosmotic conditions. In the logarithmic growth phase *nhaD *expression levels decrease for all media.

Analysis of mRNA shows an enhanced level of expression for *H. elongata nhaD *under conditions of a hyperosmotic shock (Fig. 8). Growth rates are similar for cultures under hypo-, iso- and hypersaline shock conditions but levels of *nhaD *mRNA rise only when the organism is subjected to a higher salinity.

## Discussion

### NhaD, an antiporter typical for marine bacteria?

Sodium proton antiporters are ubiquitous not only in *Bacteria *but also in *Archaea *and *Eucaria *[10] with *Clostridium fervidum *being the only known exception so far [9]. Therefore finding a Na^+^/H^+^-antiporter in *H. elongata *was not too surprising. Sequence comparison indicates strongly that the antiporter belongs to the NhaD family (Fig. 1). Highly conserved and proposedly crucial residues Asp(369) and Thr(370), which correspond to positions 288 and 289 in [20], and other conserved polar amino acids [21] are present in the *H. elongata *protein.

The interesting fact is that most antiporters of this family known so far are either found in marine bacteria belonging to the γ-subgroup of *Proteobacteria*. Haloalkaliphilic *A. amylolytica *and Lake Bogoria Isolate 25B1 might be regarded as exceptions, but we have to keep in mind that marine habitats have a pH of about 8.2 and are therefore also slightly alkaliphilic. Thus, one might speculate whether the NhaD-type antiporter is specific for marine bacteria and possibly represents a special mechanism to adapt to moderate salinities of marine habitats. Complementation and expression studies, which are discussed in detail later, do not support this. We do not observe an enhanced salt tolerance for *E. coli *wildtype (MM63 with 4% NaCl (w/v) (666 mM) and 37°C, data not shown) and we observed enhanced expression of NhaD only after a hyperosmotic shock to salinities well above those of sea water (Fig. 8). Nevertheless, this is no contradiction to NhaD being specific for marine bacteria. We have to take into consideration coastal areas which can dry out periodically, thus resulting in higher salinities than the usual 3% (w/v) (500 mM) for seawater. In such situations it could prove useful to express a special system to extrude Na^+ ^from the cytoplasm to support adaptation to salinities above 3%.

Further physiological data support the theory that NhaD is typical for marine or haloalkaliphilic habitats but indicate a different function.

### Complementation of antiporter deficient *E. coli*

As stated above, we chose to express the protein starting with the ATG at position 803 rather than 589 (Fig. 2) for two reasons. Firstly expression under control of the putative *housekeeping *promoter would yield this product and secondly the amino acid sequence reported for *V. parahaemolyticus *[12] and Fig. 1), is an equivalent to the shorter protein and completely functional. A similar observation was made by Ostroumov and her group [20] for *V. cholerae *NhaD who also successfully expressed a shorter ORF but gained a functional protein.

We also preferred using the pUC18 *lac *promoter for expression control rather than the putative promoter sequences upstream of the ORF because the promoter seems to be only induced at a reasonable level when the organism undergoes a hyperosmotic shock (Fig. 8) and also because we did observe that not all *H. elongata *promoters do work in *E. coli *(unpublished).

Complementation with *H. elongata *NhaD did not restore the lithium and sodium tolerance to the level of a wild type *E. coli *(Fig. 4). This might be due to incompatibility of this system to the host or simply due to lower numbers of antiporters in comparison to the wild type. The fact is that under given conditions NhaD does only poorly replace the NhaA/NhaB system, but nevertheless, we observed significantly enhanced growth when the antiporter was expressed in NhaA and NhaB deficient strains EP432 (Fig. 5, Tab. 2) and KNabc (Fig. 6, Tab. 3). Especially KNabc does not show any growth at 3% salinity (w/v) (500 mM) in mineral medium MM63 but has a reasonable growth rate when complemented (Fig. 6, Tab. 3). Effects are similar for EP432 in MM63 (Fig. 5A). This demonstrates that *Halomonas elongata *NhaD can replace the *E. coli *sodium/proton antiporters at elevated salinities.

To our surprise growth was equally poor in complex medium (Fig. 5A, Tab. 2) for both the control and the complemented EP432 at no (0% (w/v)) and elevated (3%) salinity (500 mM). We attribute this to the high amount of Na^+^-co-transported substarates in LB medium. A less efficient or incompatible export system for Na^+ ^might consume more energy, which could otherwise be used for growth, or might result in a high intracellular sodium concentration which damages the organism. Again, this shows that NhaD is only a poor replacement for the A/B system.

### nhaD expression in H. elongata

Quantification of mRNA indicates that we have a certain *housekeeping *level of *nhaD *expression at all salinities. This basic level is enhanced by a factor of four within a few minutes after a hyperosmotic shock (Fig. 8). Considering that we worked with a starting salinity above that of sea water and ended up doubling this salinity, as discussed above this indicates that NhaD is not needed in large amounts at marine salinities. The expression level seems to drop after one hour in a fresh culture medium, even below the starting level and not depending on shock conditions, but since we had to use 16S rRNA as a standard, this is easily explained with variations in rRNA levels at those later time points [22].

These findings comply with the two putative promoter structures upstream of the open reading frame (Tab. 1). One shows significant similarity to *sigma*70 dependent *housekeeping *promoters [23] which are also reported for *H. elongata *([24] and recent unpublished data). The other one is reasonably similar to *rpoH *dependent stress promoters. For *V. parahaemolyticus *also two putative promoters are given [12], but here only two *sigma*70-like structures were found. So far no further expression studies for NhaD type sodium/proton-antiporters are reported, this definitely needs deeper investigation.

### NhaD a Na^+^-import system?

Several publications reporting Na^+ ^export activity (e.g. [21,17]) and our own observations notwithstanding, we suppose that NhaD is rather a sodium import system than an export system. This agrees with an idea of active Na^+ ^import [16], though we disagree with the hypothesis that NhaD is a pathogenicity marker. We do not see concomitance of pathogenicity and NhaD, especially with regard to *H. elongata *and the haloalkaliphiles.

In accordance with others (e.g. [12]) we find NhaD can partially compensate for the NhaA/NhaB system in a deficient organism, but as can be seen from the physiological data in this study, it is only a poor replacement. NhaD does also not confer enhanced salt tolerance on a non-halotolerant organism. In addition to this, by expressing NhaD in an *E. coli *which still has both antiporters A and B, Li^+ ^sensitivity is increased (Fig. 7, Tab. 5, Tab. 6). One might argue that this is caused by Li^+ ^leaking through NhaD, but leakage would also occur through NhaA, NhaB and other channels and porines. Thus, leakage through one additional antiporter would not explain the drastic effect observed.

This sensitivity under conditions of heterologous expression complies with observations from *Vibrio *NhaD deletion mutants [16], which are *less *susceptible to lithium under alkaline conditions. (This referring to unpublished results only, no supporting data are given and no further data are published yet.) Both findings indicate import of small monovalent cations via NhaD.

Active Na^+ ^import would make sense on a regulatory level, but this is highly speculative especially when we take riboswich elements into consideration (Reviewed in [25]). Here we touch a field of regulatory mechanismns on a level of interactions of nucleic acids with small metabolites which we only begin to understand in detail.

## Conclusion

Although kinetics are yet to be analyzed, with the results discussed in this paper we give strong evidence that *nhaD *encodes for a functional sodium/proton-antiporter in *H. elongata*. Sequence comparison clearly indicates that this antiporter belongs to the NhaD type, which is so far found exclusively in cell membranes of marine/haloalkaliphilic γ-proteobacteria. Even an extensive database search does not yield any known or putative (transport) protein or open reading frame with significant similarity to classify for this type. This is true for other groups of *Bacteria *as well as for *Archea *or *Eucaria*. Therefore, this is definitely an indicator for NhaD being part of the adaptation mechanism for marine/haloalkaline habitats. Further research has to be done to clarify the exact role of this antiporter type. Since NhaD is probably never found as the sole antiporter of an organism this might prove an arduous task.

Identifying genes encoding for NhaA type or further antiporters in *H. elongata *may give additional support to this thesis. This might also help understanding why *H. elongata *and probably other halophilic or marine organisms have a higher lithium tolerance and may even use lithium instead of sodium for a minimum requirement of cations in the growth medium, especially since NhaD seems to be responsible for Na^+ ^(and Li^+^) import.

Considering the results so far we propose that NhaD is a system needed in a relatively low amount under housekeeping conditions but with enhanced levels of expression under shock and/or stress conditions. Especially an enhancement of Lithium sensitivity indicates that NhaD is rather a Na^+^-import system and might be involved in activation of Na^+^-sensitive promoters or control of riboswitches during osmoadaptation.

## Methods

### Organisms and cultivation

Organisms are shown in Tab. 7 with genotype and references included. If not stated otherwise *Halomonas elongata *DSM2581^T ^was cultivated at 37°C and pH 7.3 in complex medium K [26] and modified minimal medium MM63 [27], using glycerol as carbon source. *E. coli *strains were grown in modified Luria-Bertani (LB) [28] and MM63, also at 37°C and pH 7.3. Salinity and LiCl content of media were modified as necessary. For experiments concerning the range and pH dependency of Li^+^-tolerance a Tris/HCl-buffered version of MM63 with low (25 mM) phosphate content was used to prevent lithium-phosphate precipitation at high Li^+^-concentrations.

**Table 7:** Bacterial strains used in this study

Strain	Genotype	reference
*Escherichia coli *DH5α	F^- ^∅80d*lac*ZΔM15Δ(*lac*ZYA-*arg*F)U169 *rec*A1 *end*A1 *hsd*R17 (r_k_^- ^m_k_^+^)*deo*R *sup*E44 λ^- ^*thi*-1 *gyr*A96 *rel*A1	[36]
*Escherichia coli *DH5α pUCHelNhaD	see above, plus *nhaD *on pUC18 under regulation of *lac*^*I*^	This study
*Escherichia coli *EP432	*mel*BLid, Δ*nha*B1Δ*nha*A1, Δ*lac*ZY, *thr*1	[11]
*Escherichia coli *EP432 pUCHelNhaD	see above, plus *nhaD *on pUC18 under regulation of *lac*^*I*^	This study
*Escherichia coli *Knabc	TG1 *nhaA*::Km^R ^*nhaB*::Em^R ^*chaA*::Cm^R^	[12]
*Escherichia coli *KNabc pUCHelNhaD	see above, plus *nhaD *on pUC18 under regulation of *lac*^*I*^	This study
*Escherichia coli *NM81	*mel*BLid, *nha*B^+^Δ*nha*A1, kan^+^, Δ*lac*ZY, *thr*1	[13]
*Escherichia coli *NM81 pUCHelNhaD	see above, plus *nhaD *on pUC18 under regulation of *lac*^*I*^	this study
*Escherichia coli *XL1blue	*rec*A1 *end*A1 *gyr*A96 *thi hsd*R17 (r_k_^- ^m_k_^+^) *sup*E44 *rel*A1 λ^-^*lac*^- ^[F' *pro*AB *lac*I^q^ZΔM15 Tn10(tet)]	[37]
*Halomonas elongata *DSM2581^T^	Wild type	[1]

*E. coli *strains NM81 and EP432 were kindly provided by Prof. Dr. E. Padan.

*E. coli *strain KNabc was kindly provided by Prof. Dr. T. Tsuchia

To pUC18 and pUCHelNhaD bearing strains we added 50 μg/ml ampicillin and 100 mM IPTG to induce *nhaD *expression (or as control). Cultivation was done in 250 ml shaking flasks containing 50 ml medium at 180 rpm or on solid medium containing 1.5% (w/v) agar. Growth was tracked in flasks equipped for optical density (OD) measurements. OD was determined at 600 nm and corrected according to the method of Dalgaard et al. [29].

### Sequence determination

Part of the sequence was found using wobble primers (5'-GAC GTC GCC GTC GSC GTS ATC ATH-3' and 5'-GGC GAA GGC GAT GAT SAG GAA RTY-3', with H = A, C or T; R= A or G; S= G or C; Y= C or T) in a touchdown PCR on *H. elongata *genomic DNA. Sequence information was completed using standard chromosome walking techniques and inverse PCR. Sequencing reactions were carried out by GATC (Konstanz, Germany).

### Complementation of antiporter deficient *E. coli*

*H. elongata nhaD *was amplified by PCR using *Pwo *DNA polymerase (Roche) and primers HeNxpF1 (5'-GAAACTGCAtATGCGCAAGTCC-3') and HeNxpR1 (5'-CGCACGACgtcGGCTGGAAG-3'). In HeNxpF1 a *Nde*I restriction site (underlined) was introduced (C changed to t, lower case) and likewise in HeNxpR1 an AatII site. (TGG to gtc). The β-lactamase gene in pUC18 was replaced *inframe *by *H. elongata nhaD *for expression under control of the *lac*-operator yielding vector pUCHelNhaD (Fig. 3). *E. coli *DH5a and XL1 blue were used for cloning purposes, strains NM81, EP432 and KNabc were then transformed with the plasmid. pUC18 was used as a control.

### Expression analysis

RNA was extracted from *H. elongata *culture samples with the E.Z.N.A.™ Bacterial RNA kit (Peqlab). cDNA was constructed according to the protocol of Bertioli and Burrows [30]. *nhaD *cDNA was quantified by real-time PCR in a GeneAmp^® ^5700 System (Applied Biosystems) measuring SYBR^®^-Green fluorescence.

### Computer methods

Data base research was done using the NCBI Blast interface [31]. Multiple sequence alignment was carried out with MULTALIN [32] and CLUSTHALW [33]. Promoter analysis was done using the SEARCHLAUNCHER interface [34]. Hydropathy plots were also done via SEARCHLAUNCHER according to the method of Eisenberg et al. [35].

## Authors' contributions

Matthias Kurz worked on the sequence and most of the physiological experiments including expression and promoter analysis. Anika Brünig constructed the vector pUCHelNhaD and helped in the complementation experiments. Erwin Galinski helped in the conception and design of the experimental setup and in data analysis.
